# A High-Density EEG Investigation into Steady State Binaural Beat Stimulation

**DOI:** 10.1371/journal.pone.0034789

**Published:** 2012-04-09

**Authors:** Peter Goodin, Joseph Ciorciari, Kate Baker, Anne-Marie Carrey, Michelle Harper, Jordy Kaufman

**Affiliations:** Brain and Psychological Sciences Research Centre, Swinburne University of Technology, Hawthorn, Victoria, Australia; RAND Corporation, United States of America

## Abstract

Binaural beats are an auditory phenomenon that has been suggested to alter physiological and cognitive processes including vigilance and brainwave entrainment. Some personality traits measured by the NEO Five Factor Model have been found to alter entrainment using pulsing light stimuli, but as yet no studies have examined if this occurs using steady state presentation of binaural beats for a relatively short presentation of two minutes. This study aimed to examine if binaural beat stimulation altered vigilance or cortical frequencies and if personality traits were involved. Thirty-one participants were played binaural beat stimuli designed to elicit a response at either the Theta (7 Hz) or Beta (16 Hz) frequency bands while undertaking a zero-back vigilance task. EEG was recorded from a high-density electrode cap. No significant differences were found in vigilance or cortical frequency power during binaural beat stimulation compared to a white noise control period. Furthermore, no significant relationships were detected between the above and the Big Five personality traits. This suggests a short presentation of steady state binaural beats are not sufficient to alter vigilance or entrain cortical frequencies at the two bands examined and that certain personality traits were not more susceptible than others.

## Introduction

Externally produced modulation of cortical frequencies can be witnessed via electrophysiological recordings using simple stimuli such as light oscillating at a stable frequency [1] or acoustics such as human speech or consistent tones [2], [3]. Known as the Frequency Follow Response (FFR) [4], it has been suggested that numerous physiological and psychological processes can be altered through external means, effectively entraining the brain to synchronize neural activity with the stimuli (see [5] for a recent review of existing literature). Currently there are few studies that examine the role individual differences may play in affecting entrainment. [6], [7], [8], [9]

The Five Factor Model (FFM) of personality [10] identifies five traits of personality; Neuroticism (N), Extraversion (E), Openness to Experience (O), Agreeableness (A) and Contentiousness (C) and has been subject to a large amount of study examining possible electrophysiological relationships [11], [12], [13]. In a study by Stough et al. [13] photic driving was used to examine correlates of personality traits and entrainment. They found the personality traits O and C were associated with increased Theta and Beta activity across the cortex, while A showed a positive correlation in left central and temporal areas. Unfortunately no baseline period was used, so the results could be due to naturally higher levels of oscillatory activity in some personality traits, regardless of any task or stimulus.

It is currently unknown whether cortical activity is alterable through the FFM using auditory stimuli to generate entrainment. One such method purported to generate cortical entrainment are binaural beats. Binaural beats occur when two sinusoidal waves at slightly differing frequencies are presented separately to each ear [14], which are then generally experienced as a pulsating auditory sensation at the difference of frequency between the two waves. For example, a standard tone 400 Hz played to the left ear and a carrier tone of 407 Hz played to the right ear would produce the sensation of a 7 Hz binaural beat. Binaural beats are best perceived when the carrier and standard tone frequencies are at approximately 400 Hz with differences between the two frequencies of no more than 35 Hz [3].

Studies have shown a neurological basis of binaural beats perception which have assisted in identifying subcortical regions associated with processing phase differences between sounds. These have been found to be generated by neurons in the inferior colliculus, auditory cortex [15], [16] and the medial olivary nucleus, all of which are thought to be involved in processing and integration of auditory stimuli [17]. The effect of binaural beats on psychological and biological aspects however has been somewhat less clear.

A study examining binaural beat alterations on neuropsychological factors [18] found Theta (7 Hz) stimulation binaural beats to have a disadvantageous effect on memory in for the form of immediate recall of words, while an examination into the use of binaural beats as a potential treatment for anxiety failed to produce statistically significant results [19]. The use of downward frequency cycling binaural beats from Alpha to Delta as a treatment for insomnia [20] did find there was a significant increase in patient relaxation but suggested this may have been due to the calming music that typically accompanies commercially purchases binaural beat stimulation packages. A study into vigilance modulation using binaural beats by Lane et al. [21] found that Beta frequency binaural beats improved attention, resulting in participants correctly identifying more targets and fewer false alarms over a 30-minute period than those stimulated by Delta/Theta frequencies. Studies examining electromagnetic changes generated by binaural beat entrainment have yielded inconsistent results. Some [7], [16], [22], [23] have reported increases in spectral density at Theta frequency using both event related and steady state presentation methods, while others [8], [18] have failed to find evidence of the FFR using steady state presentation.

Based on the above, this study had three main aims:

1. To examine the effect of binaural beat stimulation on vigilance in a zero-back task,
2. To determine if cortical frequency entrainment is possible using steady state binaural beats, and
3. To partially replicate and extend Stough et al. [13] to determine if personality type affects baseline cortical frequencies and binaural beat entrainment.

As Stough et al. found that Beta and Theta frequencies were correlated with personality traits and alterations in vigilance; the current investigation employed these same frequencies for analysis. We hypothesised that, when undergoing stimulation by the Beta frequency carrier tones, participants would show increased vigilance as evidenced by faster reaction times to stimuli and there would be increased Beta and Theta cortical frequency power during binaural beat stimulation. It was also hypothesised that the magnitude of scores in O and C would be positively correlated with perceptual speed and overall cortical power of Theta and Beta frequencies. Finally, we hypothesised that those who scored higher in A would show higher Beta frequency power in the left temporal and central cortical areas. No specific hypotheses were made relating to baseline periods, Neuroticism or Extraversion and entrainment.

## Methods

### Participants

Forty-five participants were initially recruited from the general public and from a Melbourne university community. Exclusion criteria for the study consisted of any reported neurological disorder or known hearing damage or loss. Data from 14 participants was removed from the study due to file corruption, missing electrophysiological or behavioural data, (4 females, 3 males), excessive artifact on the EEG (2 females, 3 males) and reports of not being able to perceive the binaural beats (1 female, 1 male). This left a total cohort of 31 participants in the study (20 females, 11 males) with ages ranging from 18 to 60 (*M* = 28.90, *S.D.* = 10.82). Four were left-handed (3 female, 1 male). Written informed consent was obtained from all participants involved in the study; which was approved by Swinburne University's Human Research Ethics Committee.

### Materials

Participants' personality trait scores were measured using the NEO Five Factor Inventory (NEO FFI) questionnaire [24]. The NEO FFI consists of 60 items measuring personality traits across 5 domains (Neuroticism, Extraversion, Openness to Experience, Agreeableness and Conscientiousness) on a 5-point Likert scale (1 = Strongly Disagree, 5 = Strongly Agree) with a theoretical range for each personality trait between 15 and 60. Internal consistency and test-retest reliability have been found adequate.

Binaural beats at 7 Hz (Theta) and 16 Hz (Beta) were produced using BrainWave Generator software version 3.1.12 (http://www.bwgen.com/). A 400 Hz standard tone and a 407 Hz for Theta or 416 Hz for Beta carrier tone were used to attempt entrainment. All stimuli were played to participants at 70 dB SPL, which is within the range recommended by Stevens et al. [8] to induce Theta entrainment.

All binaural beat tones were two minutes in length, with a total of 4 minutes continued presentation while white noise baseline was presented between binaural beat stimulation epochs. Two minutes of constant stimulation per ear was deemed an acceptable timeframe to detect entrainment as studies applying binaural beats for less than two seconds have detected event related potential modulation [16], [23], while the results of a recent steady state study [22] suggest entrainment happens within seconds of the binaural stimulation being presented. The waveforms were inspected for imperfections that could distort the tones using the freeware audio editing program Audacity version 1.2.6 (http://audacity.sourceforge.net/).

The experimental procedure was presented using E-Prime version 2.0.8.22 on a Dell Optiplex 755 computer. A common paradigm used in neuroimaging [25], [26], [27], [28], [29], the 0-back task was used as a measure of vigilance and to keep participants in an approximately similar state of arousal. The tones used to elicit a binaural beat were played through a pair of stereo headphones positioned so the earpieces and headband did not press against electrodes and were stable while participants engaged in the vigilance task. A computer keyboard was used to register participant's response times (in milliseconds) to the 0-back task target stimuli as well as incorrect answers and the onset of the binaural beat.

### EEG recording and Electrode Placement

**Figure 1:**
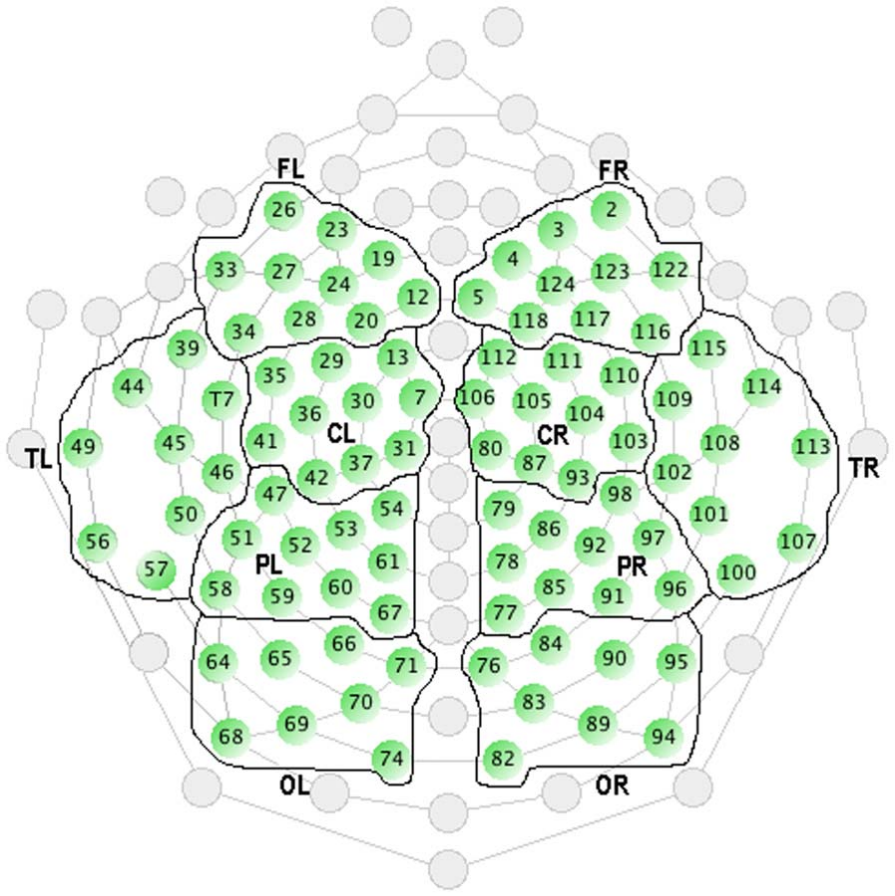
Included Electrodes by Cortical Area. Note: FL = Front Left, FR = Front Right, CL = Central Left, CR = Central Right, TL = Temporal Left, TR = Temporal Right, PL = Parietal Left, PR = Parietal Right, OL = Occipital Left, OR = Occipital Right.

EEG data was collected using an Electrical Geodesics Inc. (EGI) EEG acquisition system consisting of a 128-channel Hydrocel Geodesic Sensor Net and a Net Amps 300 high-impedance amplifier. The data were recorded using EGI's acquisition software (i.e., Net Station version 4.2.4) on an Apple Mac Pro computer running OS 10.4. Impedances were kept at less than 50 kΩ as per the manufacturer's instructions (Electrical Geodesics, Inc, Eugene, OR) and examined for electrode bridging. The EEG signal was sampled at 500 Hz. Figure 1 illustrates the location of electrodes for high density EEG recording and the allocations to specific anatomical regions for data reduction.

### Pre-experiment and Training

Participants were seated in a quiet recording room and asked to complete a demographic questionnaire which included items on gender, age and handedness. The participant's head was measured for the appropriate electrode net size and an audiometric test was performed. During the test, participants were played a tone over a 10-second period that alternated from the left to right channel at the 5-second mark. The tone consisted of a 400 Hz sinusoidal waveform and was played at 70 dB SPL.

Following the audiometric test, participants listened to a 30-second example of binaural beat recording at alpha frequency (13 Hz) to minimize any potential interactions on the frequencies of interest. Participants then completed the NEO FFI questionnaire. After completion of the questionnaire the electrode net was place on the participant's head and secured as suggested by the manufacturer's instructions (Electrical Geodesics, Inc, Eugene, OR).

### Experiment

**Figure 2:**
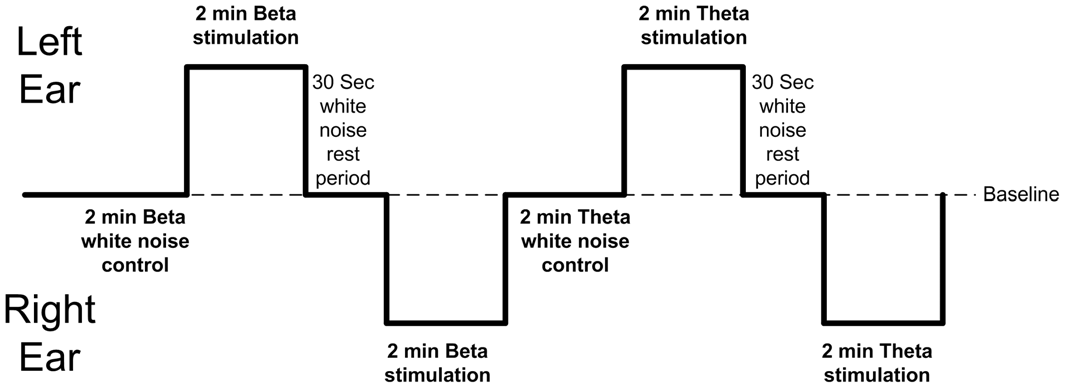
Experimental Protocol and Timings.

The experiment consisted of eight epochs, with four binaural beat presentations; two of Beta (16 Hz) and Theta (7 Hz) frequencies to left and right ears respectively (See Figure 2). The epochs consisted of a two-minute white noise control period, two minutes of a binaural beat carrier tone played to the left ear, a 30-second white noise rest period, two minutes of a binaural beat carrier tone played to the right ear, a second two minute white noise control period then a repeat of the earlier epochs, but playing the alternative frequency. The total running time was 13 minutes per participant. EEG was recorded concurrently during all epochs. The order in which binaural beat frequency stimulation occurred was counterbalanced, with approximately half the participants receiving Beta frequency stimuli first.

In order to maintain engagement, test vigilance and control for eye movement artifact, participants were asked to do two consecutive tasks during the recording session. The first was a continuous 0-back task similar to that described in [21]. Capital letters appeared centrally on a screen every five seconds and participants were asked to press a key when the target letter, a capitalized M appeared. N was not used in the task due to its similar appearance to the target letter. In total, 29 target presentations over the 13-minute experiment occurred. During the binaural beat stimulation periods, 10 targets occurred during Beta stimulation (6 during left binaural beat carrier tone) and 11 during Theta stimulation (6 during left binaural beat carrier tone The second task required participants to indicate when they perceived the binaural beat by pressing another key. Their reaction time relative to the start of the stimulation epochs were recorded and used for analysis. Participants were given the length of the stimulation epochs (two minutes) to answer and instructed to only press the key when they were aware of the beat. If they were unable to detect the beat, they were instructed not to push the key. In addition to the key presses related to binaural beat perception, upon completion of the experiment participants were explicitly asked if they perceived the beats at any point during the study. Participants indicating they did not hear the beats (n = 2) were excluded from further analysis.

### EEG Analysis

The recorded EEG was analysed using Brain Vision Analyzer version 2.

Data was converted from Net Station format to Brain Vision Analyzer format via an EEGLAB plug-in. The data were filtered using a low cut of .05 Hz (12 dB per octave) and a high cut of 35 Hz (48 dB per octave) with an averaged electrode reference. All participant data was segmented into blocks relative to the condition presentation time and corrected for EOG using the Gratton, Coles and Donchin [30] method. A Fast Fourier transformation was applied and the absolute spectral power for the two frequency ranges for each electrode were calculated (5.5 Hz to 7.5 Hz for Theta band and 15.5 to 17.5 for Beta band). Data reduction was employed for exploratory purposes; the electrodes were grouped into the major left and right cortical lobes (frontal, central, parietal, temporal and occipital), with each area containing between eight and ten electrodes. Electrodes at the extremes of the net were culled from the analysis due to their potential for high levels of EOG and EMG artifact. The average spectral powers for the two bands were calculated for each group.

### Personality Scoring

The NEO FFI questionnaire scores were compiled and calculated using a Teleform scanner. NEO FFI scores were divided into low, average and high to examine possible effects of personality trait strength. Categories were created by calculating the means and standard deviations for each personality trait and split according to one standard deviation. A score of low for each of the personality traits were one standard deviation below the mean or less, while a score of high was one standard deviation above the mean or more. Additionally, as the FFM is thought of as a continual construct, NEO FFI scores were also kept as non-categorical, interval data for correlational analysis.

### Reaction Time Data

Individual binaural beat stimulation reaction time data was calculated by taking the mean of all target letters that appeared during the binaural beat conditions (for example, left Beta carrier tone, right Beta carrier tone) while mean Beta and Theta reaction time data was calculated from left and right reaction times in the particular frequency range. Baseline reaction times were calculated from target stimuli presented during the two white noise conditions.

## Results

In order to increase statistical power, the counterbalanced groups were collapsed into a single collection. Chi square tests revealed no significant difference between the groups for sex (χ ^2^(1, N = 31) = 2.203, *p* = .258) or handedness (χ ^2^(1, N = 31) = 1.651, *p* = .304). One-way analysis of variance (ANOVA) examination determined no significant differences between the counterbalanced groups for age (*F*(1,29) = .05, *p* = .811 NEO trait scores (N (*F*(1,29) = .24, *p = *.628), E (*F*(1,29) = 2.22, *p* = .146), O (*F*(1,29) = .22, *p* = .640), C (*F*(1,29) = 1.69, *p* = .204), A (*F*(1,29) = .70, *p* = 409)), binaural beat perception reaction time (Beta frequency (*F*(1,29) = .01, *p* = .939), Theta frequency (*F*(1,29) = .22, *p* = .636)), control spectral power (Beta frequency (*F*(1,29) = .01, *p* = .949), Theta frequency (*F*(1,29) = .69, *p = *.410)), experimental spectral power (Beta frequency (*F*(1,29) = .04, *p* = .837), Theta frequency (*F*(1,29) = 3.94, *p* = .057) or the reaction times from the vigilance task conditions (binaural beat off (*F*(1,29) = 2.86, *p* = .101), averaged over frequencies binaural beat on (*F*(1,29) = 1.32, *p* = .259), Beta frequency on (*F*(1,29) = 3.16, *p* = .086), Theta frequency on (*F*(1,29) = .13, *p* = .718). NEO group membership numbers are shown below in Table 1. There was an approximately normal distribution across all five personality domains.

**Table 1:** Neo Group Membership including total numbers and percentages.

NEO Group	Range	Total	Percentage %
Neuro	Low	9	29
	Average	16	51.6
	High	6	19.4
Extra	Low	4	12.9
	Average	22	71
	High	5	16.1
Open	Low	5	16.1
	Average	22	71
	High	4	12.9
Consc	Low	2	6.5
	Average	25	80.6
	High	4	12.9
Agree	Low	3	9.7
	Average	24	77.4
	High	4	12.9

N = 31.

Note: Neuro = Neuroticism, Extra = Extraversion, Open = Openness to Experience, Consc = Conscientiousness, Agree = Agreeableness.

To examine if binaural beats affected vigilance, paired samples t-tests were conducted. The tests did not reveal any significant difference between left Beta carrier tone stimulation and right Beta carrier tone stimulation reaction times (*t*(30) = .540, *p = *.593), Beta and Control reaction times (*t*(30) = .84, *p = *.403), Beta and Theta stimulation reaction times (*t*(30) = 1.12, *p* = .272), left Theta carrier tone stimulation and right Theta carrier tone stimulation reaction times (*t*(30) = .995, *p* = .347) or Theta stimulation and Control times (*t*(30) = 1.03, *p* = .310). This suggests vigilance as measured by reaction times to a 0-back task was not altered with beat binaural beat stimulation of either Beta or Theta frequencies, contrary to the hypothesis. The means and standard deviations of the conditions are shown below in Table 2.

**Table 2:** Means and Standard Deviations of Reaction Time (ms) in the Vigilance Task during Beta binaural beat stimulation, Theta stimulation and Control conditions.

Condition	Reaction Time Mean (S.D.)
Beta Left Carrier	645.17 (317.39)
Beta Right Carrier	615.22 (133.61)
Theta Left Carrier	582.35 (125.23)
Theta Right Carrier	619.32 (254.54)
Beta	643.52 (279.67)
Beta Control	629.47 (297.13)
Theta	596.42 (154.98)
Theta Control	631.95 (162.15)

N = 31.

Correlational analysis was employed to determine if there were interrelationships not detected by the t-tests due to the formation of three categorical groups. NEO personality traits and reaction times in the vigilance task were examined on a continuous dimension, however no significant results between the two were found (Table 3). There were significant positive correlations found between C and N (*r* = .45, *p* = <.01), C and E (*r = *.45, *p* = <.05), Theta and Beta binaural beat stimulation reaction times (*r = *.39, *p* = <.01) and Control reaction times during Theta (*r = *.49, *p* = <.01) and Beta (*r* = .91, *p* = <.01) conditions. These results suggest that personality traits as measured by the NEO do not alter susceptibility to binaural beat induced increases in vigilance.

**Table 3:** Correlation Table of NEO Personality Traits and Vigilance Task Reaction Time during Beta, Theta and Control Conditions.

	Neuro	Extra	Open	Consc	Agree	BetaRT	BContRT	ThetaRT	TContRT
Neuro	1.00								
Extra	−0.54**	1.00							
Open	−0.34	0.18	1.00						
Consc	.46**	.41*	0.25	1.00					
Agree	−0.24	0.31	0.04	0.15	1.00				
BetaRT	0.10	−0.03	−0.23	−0.07	0.08	1.00			
BContRT	.207	−.151	−.238	−.189	.022	.951**	1.00		
ThetaRT	0.26	−0.19	−0.31	−0.23	0.05	.39*	0.45	1.00	
TContRT	−.144	.200	−.124	.173	.154	.516**	.494**	.380*	1.00

N = 31.

** . Correlation is significant at the 0.01 level (2-tailed).

* . Correlation is significant at the 0.05 level (2-tailed).

Note: Neuro = Neuroticism, Extra = Extraversion, Open = Openness to Experience, Consc = Conscientiousness, Agree = Agreeableness, BetaRT = Beta Reaction Time, BContRT = Beta Control Reaction Time, ThetaRT = Theta Reaction Time, TContRT – Theta Control Reaction Time.

To determine if cortical frequencies could be entrained through binaural beat stimulation, a repeated measures ANOVA was employed. No statistical differences were detected between Beta control and stimulation conditions (*F*(1,30) = 1.23, *p* = .227) or Theta control and stimulation conditions (*F*(1,30) = .24, *p* = .625). This was contrary to the hypothesis that binaural beat stimulation would affect a level of entrainment in the cortex at Beta and Theta frequencies. The means and standard deviations of cortical power can be seen in Table 4 below.

**Table 4:** Means and Standard Deviations of Cortical Power Spectral Density (in µV) by Condition.

Condition	Power Mean (S.D.)
Beta Control	15.70 (1.07)
Beta Experimental	16.55 (1.24)
Theta Control	45.23 (1.71)
Theta Experimental	44.52 (2.20)

N = 31.

To examine the viability of the hypothesis that those who scored high on the O and C personality traits would have increased Beta and Theta cortical power spectral density respectively during binaural beat stimulation, 3(Neo trait score)×2(Condition) ANOVAs were conducted for each of the personality traits and binaural beat frequencies used in the study . The table below (Table 5) shows the means and standard deviations of Beta cortical power spectral density by NEO personality traits, trait scores and conditions.

**Table 5:** Beta Cortical Power Spectral Density by Personality Trait, Trait Score and Condition.

	Power (µV) Mean (S.D.)		
Condition	Low	Average	High
Neuro Beta Control	13.79 (1.37)	16.88 (1.38)	15.43 (3.71)
Neuro Beta Experimental	13.44 (1.15)	18.31 (1.36)	16.49 (4.99)
Extra Beta Control	14.00 (1.79)	16.06 (1.45)	15.48 (1.40)
Extra Beta Experimental	14.40 (2.77)	17.36 (2.77)	14.67 (0.86)
Open Beta Control	18.46 (1.27)	14.35 (1.33)	19.70 (2.34)
Open Beta Experimental	18.51 (1.78)	15.29 (1.54)	21.01 (3.20)
Consc Beta Control	9.73 (4.80)	16.72 (1.19)	12.36 (1.47)
Consc Beta Experimental	10.72 (3.58)	17.58 (1.43)	12.96 (1.22)
Agree Beta Control	15.41 (2.60)	16.12 (1.32)	13.4 (1.37)
Agree Beta Experimental	14.87 (1.97)	17.21 (1.56)	13.83 (1.01)

N = 31.

The ANOVA found no significant interactions between white noise control and Beta stimulation condition for any of the NEO personality traits (N (*F*(2,28) = .50, *p* = .611, E *F*(2,28) = .51, *p* = .603, O *F*(2,28) = .11, *p* = .896, C *F*(2,28) = .01, *p* = .992, A *F*(2,28) = .20, *p* = .816). These results did not support the third hypothesis, as Beta power did not increase in O with Beta binaural beat stimulation compared to control. Table 6 (below) contains the means and standard deviations by condition and score for Theta binaural beat stimulation.

**Table 6:** Theta Cortical Power Spectral Density by Personality Trait, Trait Score and Condition.

	Power (µV) Mean (S.D.)		
Condition	Low	Average	High
Neuro Theta Control	50.00 (8.25)	43.62 (9.99)	42.40 (8.69)
Neuro Theta Experimental	50.64 (12.05)	40.75 (10.26)	45.40 (10.86)
Extra Theta Control	45.82 (4.92)	44.68 (10.10)	47.21 (10.86)
Extra Theta Experimental	45.43 (9.55)	44.39 (13.59)	44.37 (8.79)
Open Theta Control	45.68 (9.39)	45.25 (9.36)	44.59 (13.12)
Open Theta Experimental	45.30 (16.31)	44.55 (11.76)	43.40 (12.78)
Consc Theta Control	48.37 (10.74)	43.92 (9.61)	51.87 (6.90)
Consc Theta Experimental	61.30 (2.87)	43.31 (2.88)	49.97 (11.55)
Agree Theta Control	42.18 (8.81)	44.53 (9.81)	51.72 (6.76)
Agree Theta Experimental	42.97 (10.02)	43.66 (12.51)	50.88 (12.67)

N = 31.

The statistical analysis conducted found no significant interactions between the NEO traits, score or Theta stimulation condition for N (*F*(2,28) = 1.39, *p* = .265), E (*F*(2,28) = .20, *p* = .820), O (*F*(2,28) = .01, *p* = .989) or A (*F*(2,28) = .055, *p* = .947). There was a significant interaction between C and Theta binaural beat stimulation on Theta frequency power (*F*(2,28) = 3.67, *p* = .038), however this result failed to survive a post-hoc Student-Newman-Keuls test (*p* = .243). These results also did not support the hypothesis as cortical Theta power levels in C did not differ from the control period.

Examination of the fourth hypothesis was conducted using a correlational analysis as performed in the study by Stough et al. Correlations for personality traits and cortical spectral density power in both Beta and Theta stimulation conditions in the temporal-central region are shown in Table 7.

**Table 7:** Correlations of Beta, Theta Experimental and Control Frequency Binaural Beat Cortical Powers in Divided Cortical Areas and NEO Personality Traits.

		Neuro				Extra				Open				Consc				Agree			
Condition		Beta	BCon	Theta	TCon	Beta	BCon	Theta	TCon	Beta	BCon	Theta	TCon	Beta	BCon	Theta	TCon	Beta	BCon	Theta	TCon
Frontal	Left	0.12	0.04	−0.23	−0.25	−0.10	−0.04	0.04	0.02	−0.07	0.05	0.05	0.27	−0.30	−0.24	−0.01	0.22	−0.06	−0.15	0.23	0.19
	Right	0.21	0.17	−0.24	−0.27	−0.23	−0.15	0.12	0.04	−0.17	0.02	0.05	0.10	−0.31	−0.22	0.07	0.26	−0.10	−0.24	0.20	0.15
Central	Left	0.14	0.07	−0.04	−0.14	−0.10	−0.01	−0.10	−0.05	−0.08	0.06	−0.05	0.01	−0.32	−0.15	−0.07	0.11	−0.09	−0.23	0.21	0.29
	Right	0.18	0.16	−0.05	−0.28	−0.18	−0.13	−0.03	−0.01	−0.18	0.00	−0.02	−0.01	−0.30	−0.21	0.03	0.26	−0.15	−0.24	0.16	0.11
Temporal	Left	0.23	0.12	−0.01	−0.17	−0.09	0.01	−0.04	0.07	−0.20	−0.01	0.07	−0.04	−0.18	−0.03	−0.10	0.14	−0.03	−0.24	0.20	0.29
	Right	0.11	0.09	−0.10	−0.37	−0.06	−0.04	0.04	0.13	−0.12	0.13	0.07	0.03	−0.18	−0.18	−0.09	0.05	−0.06	−0.14	0.24	0.23
Parietal	Left	0.25	0.19	−0.20	−0.26	−0.22	−0.19	0.16	0.32	−0.21	−0.02	0.06	0.08	−0.24	−0.18	0.08	0.27	−0.13	−0.32	0.16	0.24
	Right	0.24	0.18	−0.08	−0.27	−0.22	−0.19	0.08	0.13	−0.17	0.13	−0.01	0.07	−0.24	−0.23	0.01	0.21	−0.19	−0.28	0.25	0.13
Occipital	Left	0.23	0.16	−0.04	−0.21	−0.09	−0.02	−0.02	0.18	−0.21	−0.02	0.10	−0.16	−0.24	−0.17	−0.09	0.21	−0.08	−0.29	0.14	0.19
	Right	0.10	0.05	−0.13	−0.37	−0.05	0.01	0.04	0.16	−0.13	0.09	0.05	−0.08	−0.20	−0.20	−0.09	0.07	−0.05	−0.12	0.24	0.22

N = 31.

* = Significant to 0.05 (Two tailed).

** = Significant to 0.01 (Two tailed).

Note: Neuro = Neuroticism, Extra = Extraversion, Open = Openness to Experience, Consc = Conscientiousness, Agree = Agreeableness, BCon = Beta Control, TCon = Theta Control.

As can be seen from the table, no significant correlations were detected between the NEO traits, cortical area or spectral density powers at Control, Beta or Theta frequencies. This did not support the hypothesis that increases in A score would show increases in Beta power in the left temporal-central cortical areas. It also suggests that personality traits as measured by the FFM do not increase or decrease the power of cortical frequencies in either the Beta or Theta range.

## Discussion

This study aimed to determine if binaural beats presented at frequencies corresponding to Beta (16 Hz) and Theta (7 Hz) could alter the cognitive faculty of attention through a vigilance task, if they could be used to entrain the brain to their particular resonance, and if personality traits as measured by the NEO FFI mediated either of the previous variables. Four hypotheses were formulated based on previous findings in order to test the aims; that Beta frequency stimulation would assist in sustaining vigilance, binaural beat stimulation at either Beta or Theta frequencies would increase overall cortical power at those frequencies, that the personality traits of O and C would show increased susceptibility to Beta and Theta beat entrainment respectively and that A would show increased power in Beta frequency in the left temporal-central areas. Statistical analysis revealed that none of the four hypotheses were supported. There were no significant changes in reaction times in either Beta or Theta stimulation or across the NEO traits, which is contrary to the results reported by Lane et al. This may be due to the frequencies in which binaural beats were presented to participants or differences in measurement of vigilance between the two studies. The current study played four minutes (two minutes per ear) of each frequency binaural beat to participants, and used 16 Hz and 7 Hz as the intended entrainment frequencies. By contrast, Lane et al. used a 30-minute task in which participants were presented with binaural beats at 1.5, 4, 16 and 24 Hz, with carrier frequencies cycling from 100 to 300 Hz and combined lower and higher frequencies into pools of Delta/Theta and Beta. Wahbeh et al. used a similar stimulation time for their study yet failed to note any entrainment, which suggests the length of stimulation may not be a factor. Therefore it may be possible the higher vigilance noted in Lane's study was due to the use of both an interaction between the 16 and 24 Hz frequency cycling binaural beats. Secondly, the tasks used to measure vigilance were not identical and may have tapped into difference cognitive domains.

This study used reaction time as an analogue for vigilance, as it was thought that those who are more engaged would be more likely to respond faster to a target than those who are not. Correct versus incorrect measures were recorded, however all included participants successfully identified the target stimuli 100% of the time, suggesting the task may have been too easy. Lane et al. used target hits to non-target false alarm key presses as their measure, which it could be argued may not just reflect vigilance but also the ability to inhibit irrelevant stimuli. Future studies may wish to design a task that adequately measures both in order to elucidate.

Entrainment of Theta frequency as evidenced by increased power spectral density was not observed in this study. Additionally increased Beta power during the stimulation period was also not observed. These results differ from those found by Brady et al., however support those found in the follow up study by Stevens et al. and Wahbeh et al. In contrast, Karino et al. found increased Theta components, however this study used event related presentations to elicit alterations in cortical frequencies.

No direct differences between personality traits and cortical power through binaural beat stimulation, or correlates between the two were detected. This did not support the results of Stough et al., who found that using photic stimulation at Beta and Theta frequencies produced higher overall cortical entrainment in those who scored higher in O and C, while A was associated with increased Theta activity in the left temporal-central area. The obvious answer for these differences would be the unlike methods of attempting entrainment, as photic driving uses pulses of light presented at a steady state in order to generate similar frequency neuronal firing where as in this study steady state binaural beats were used.

While several studies have used binaural beats in conjunction with photic driving into order to generate psychological effects; for a review see [5] there has yet to be any studies examining delineation between the two, especially for electrophysiological responses. That no significant differences were found in Beta or Theta power in any division of the cortex or for any personality trait was unexpected. It may be that this study simply failed to expose participants to binaural beat stimulation for long enough. There has been a suggestion that at least five minutes is required for observation of entrainment to occur, however the authors of this study were unable to locate the abstract or article that made this claim [31] thus not able to verify it. Other studies tend to use periods of up to 30 minutes for binaural stimulation, however given the contradictory results discussed above and that event related studies [16] managed to generate electrophysiological increases in Theta frequency using binaural beats in a period of 2 seconds, the time of presentation is not thought to be an issue. The task itself may have contributed to an inability to observe cortical frequency alteration using binaural beats, at least in the Theta range, due to the suggestion Beta activity is related to visual processing, especially in the temporo-parietal areas [32] and as such may have masked increases in Theta. However some [16] have used visual stimuli and also found evidence of frequency alteration, at least using an event related design. As such a task/no task paradigm might be investigated in the future. Examining the mean frequency power over the two-minute presentation blocks may have prevented detection of entrainment. If entrainment only occurred in very brief periods in the experimental condition rather than presumed sustained increases at the target frequencies, this study would not have been able to detect it. Instead, a frequency over time analysis is strongly recommended in future examinations to rule out the possibility of entrainment in sporadic brief events.

A final consideration is the use of pink noise, overlaid music or sound, to generate some sort of effect. One study [33] compared music with an embedded binaural beat to music without one and generated a significant decrease in pain medication both during and after an operation, however the study was not controlled as participants were allowed to choose their own music. Also, other studies using pink noise [8], [18] have not detected entrainment, but have found psychological changes previously discussed. Comparing pink noise with a binaural beat, without and a control and subsequent effects on electrophysiological and psychological factors may be of interest.

In conclusion, this study aimed to examine if binaural beats were able to alter psychological processes and entrain cortical frequencies. Furthermore it aimed to examine if personality traits modulated entrainment. No statistically significant changes or relationships were detected between binaural beat stimulation at Beta and Theta frequencies and white noise control conditions in any personality trait, the vigilance task or EEG power spectra analysis. These results suggest that relatively short presentation steady state binaural beat stimulation at Beta and Theta frequencies are insufficient to generate entrainment and in turn this lack of entrainment does not seem to be related to personality traits. Additionally it appears that short presentation stimulation of binaural beats is ineffective at altering vigilance.
