# Correction: A High-Density EEG Investigation into Steady State Binaural Beat Stimulation

**DOI:** 10.1371/annotation/89b655ea-6877-411d-abee-e1f4806f5f78

**Published:** 2012-04-13

**Authors:** Peter Goodin, Joseph Ciorciari, Kate Baker, Anne-Marie Carrey, Michelle Harper, Jordy Kaufman

There was a spelling error in the fourth author's name. The correct spelling is Anne-Marie Carey. The correct citation is: Goodin P, Ciorciari J, Baker K, Carey A-M, Harper M, et al. (2012) A High-Density EEG Investigation into Steady State Binaural Beat Stimulation. PLoS ONE 7(4): e34789. doi:10.1371/journal.pone.0034789 

